# Royal Jennerian Society

**Published:** 1806-02

**Authors:** Charles Murray

**Affiliations:** Secretary


### ROYAL JENNERIAN SOCIETY,

### For the Extermination of the Small-pox.

At a Special Meeting of the Board of Directors, held at the Central House of the Society, No. 14, Salisbury-square, Fleet-street: The Report of the Medical Council, on the Subject of Vaccine Inoculation, having been laid before the Board,

Resolved,—That the same be immediately printed under the direction of the Medical Council, and that they be requested to subjoin their individual signatures to the Report for publication. (Extract from the Minutes.)

Charles Murray, Secretary.

### REPORT.

The Medical Council of the Royal Jennerian Society, having been informed that various cases had occurred, which excited prejudices against vaccine inoculation, and tended to check the progress of that important discovery in this kingdom, appointed a Committee of twenty-five of their Members to inquire, not only into t e nature and truth of such cases, but also into the evidence respecting instances of small-pox, alleged to have occurred twice in the same person.

In consequence of this reference, the Committee made diligent inquiry into the history of a number of cases, in which it was supposed that vaccination had failed to prevent the small-pox, and also of such cases of small-pox, as were stated to have happened subsequently to the natural or inoculated small-pox.

In the course of their examination the Committee learned, that opinions and assertions had been advanced and circulated, which charged the cow-pox with rendering patients liable to particular diseases, frightful in their appearance and hitherto unknown; and judging such opinions to be connected with the question as to the efficacy of the practice, they thought it incumbent upon them to examine examine also into the validity of these injurious statements respecting vaccination.

After a very minute investigation of these subjects, the result of their inquiries has been submitted to the Medical Council; and from the Report of the Committee it appears;

1. That most of the cases, which have been brought forward as instances of the failure of vaccination to prevent the small-pox, and which have been the subjects of public attention and conversation, are either wholly unfounded or grossly misrepresented.

2. That some of the cases are now allowed, by the very persons who first related them, to have been erroneously stated.

3. That the statements of such of those cases as are published, have, for the most part, been carefully investigated, ably discussed, and fully refuted, by different writers on the subject.

4. That notwithstanding the most incontestible proofs of such misrepresentations, a few medical men have persisted in repeatedly bringing the same unfounded and refuted reports, and misrepresentations, before the public; thus perversely and disingenuously labouring to excite prejudices against vaccination.

5. That in some printed accounts adverse to vaccination, in which the writers had no authenticated facts to support the opinions they advanced, nor any reasonable arguments to maintain them, the subject has been treated with indecent and disgusting levity; as if the good or evil of society were fit objects for sarcasm and ridicule.

6. That when the practice of vaccination was first introduced and recommended by Dr. Jenner, many persons, who had never seen the effects of the vaccine fluid on the human system, who were almost wholly unacquainted with the history of vaccination, the characteristic marks of the genuine vesicle, and the cautions necessary to be observed in the management of it, and were therefore incompetent to decide whether patients were properly vaccinated or not, nevertheless ventured to inoculate for the cow-pox.

7. That many persons have been declared duly vaccinated, when the operation was performed in a very negligent and unskilful manner, and when the inoculator did not afterwards see the patients, and therefore could not ascertain whether infection had taken place or not; and that to this cause are certainly to be attributed many of the cases adduced in proof of the inefficacy of cow-pox.

8. That

8. That some cases have been brought before the Committee, on which they could form no decisive opinion, from the want of necessary information as to the regularity of the preceding vaccination, or the reality of the subsequent appearance of the small-pox.

9. That it is admitted by the Committee, that a few cases have been brought before them, of persons having the small-pox, who had apparently passed through the cow-pox in a regular way.

10. That cases, supported by evidence equally strong, have been also brought before them, of persons who, after having once regularly passed through the small-pox, either by inoculation or natural infection, have had that disease a second time.

11. That in many cases, in which the small-pox has occurred a second time, after inoculation or the natural disease, such recurrence has been particularly severe, and often fatal; whereas, when it has appeared to occur after vaccination, the disease has generally been so mild, as to lose some of its characteristic marks, and even sometimes to render its existence doubtful.

12. That it is a fact well ascertained, that, in some particular states of certain constitutions, whether vaccine or variolous matter be employed, a local disease only will be excited by inoculation, the constitution remaining unaffected; yet that matter taken from such local vaccine or variolous pustule is capable of producing a general and perfect disease.

13. That if a person, bearing the strongest and most indubitable marks of having had the small-pox, be repeatedly inoculated for that disease, a pustule may be produced, the matter of which will communicate the disease to those who have not been previously infected.

14. That, although it is difficult to determine precisely the number of exceptions to the practice, the Medical Council are fully convinced that the failure of vaccination, as a preventive of the small-pox, is a very rare occurrence.

15. That of the immense number who have been vaccinated in the Army and Navy, in different parts of the United Kingdom, and in every quarter of the globe, scarcely any instances of such failure have been reported to the Committee, but those which are said to have occurred in the metropolis, or its vicinity.

16. That the Medical Council are fully assured, that in very many places, in which the small-pox raged with great great violence, the disease has been speedily and effectually arrested in its progress, and in some populous cities wholly exterminated, by the practice of vaccination.

17. That the practice of inoculation for the small-pox, on its first introduction into this country, was opposed and very much retarded, in consequence of misrepresentations and arguments drawn from assumed facts, and of miscarriages arising from the want of correct information, similar to those now brought forward against vaccination, so that nearly fifty years elapsed before small-pox inoculation was fully established.

18. That, by a reference to the Bills of Mortality, it will appear that, to the unfortunate neglect of vaccination, and to the prejudices raised against it, we may, in a great measure, attribute the loss of nearly two thousand lives by the small-pox, in this metropolis alone, within the present year.

19. That the few instances of failure, either in the inoculation of the cow-pox, or of the small-pox, ought not to be considered as objections to either practice, but merely as deviations from the ordinary course of nature.

20. That if a comparison be made between the preservative effects of vaccination, and those of inoculation for the small-pox, it would be necessary to take into account the greater number of persons who have been vaccinated within a given time; as it is probable that, within the last seven years, nearly as many persons have been inoculated for the cow-pox, as were ever inoculated for the small-pox, since the practice was introduced into this kingdom.

21. That, from all the facts which they have been able to collect, it appears to the Medical Council, that the cow-pox is generally mild and harmless in its effects; and that the few cases, which have been alleged against this opinion, may be fairly attributed to peculiarities of constitution.

22. That many well-known cutaneous diseases, and some scrophulous complaints, have been represented as the effects of vaccine inoculation, when in fact they originated from other causes, and in many instances occurred long after vaccination; and that such diseases are infinitely less frequent after vaccination, than after either the natural or inoculated small-pox.

Having stated these facts, and made these observations, the Medical Council cannot conclude their Report upon a subject subject so highly important and interesting to all classes of the community, without making this solemn declaration:

That, in their opinion, founded on their own individual experience, and the information which they have been able to collect from that of others, mankind have already derived great and incalculable benefit from the discovery of vaccination; and that it is their full belief, that the sanguine expectations of advantage and security, which have been formed from the inoculation of the cow-pox, will be ultimately and completely fulfilled.

Signed,

Ed. Jenner, M. D. President
J. C. Lettsom, M. D. V.P.
John Ring, V.P.
Joseph Adams, M. D.
John Addington
C. R. Aikin
Wm. Babington, M. D.
M. Baillie, M. D.
W. Blair
Gil. Blane, M. D.
Isaac Buxton, M. D.
Wm. Chamberlaine
John Clarke, M. D.
Astley Cooper
Wm. Daniel Cordell
Richard Croft, M. D.
Tho. Denman, M. D.
John Dimsdale
Henry Field
Edward Ford
Joseph Fox
Will. M. Fraser, M. D.
William Gaitskell
William Hamilton, M. D.
John Hingeston
Everard Home
Robert Hooper, M. D.
Joseph Hurlock
John Jones
Tho. Key
Francis Knight
E. Leese
L. Leese
William Lewis
William Lister, M. D.
Alex. Marcet, M. D.
Joseph Hart Myers, M. D.
James Parkinson
Tho. Paytherus
John Pearson
George Rees, M. D.
John Gibbes Ridout
J. Squire, M. D.
James Upton
J. Christian Wachsell
Thomas Walshman, M. D.
Robert Willan, M. D.
Allen Williams
James Wilson
J. Yelloly, M. D.

John Walker, Secretary to the Council.

